# Factors Affecting Dental Students’ Comfort with Online Synchronous Learning

**DOI:** 10.3390/dj10020026

**Published:** 2022-02-12

**Authors:** David G. McMillan, Olivia R. Kalloo, Roberto A. Lara, Mariana Pavlova, Donna Kritz-Silverstein

**Affiliations:** 1 College of Dental Medicine, Roseman University of Health Sciences, South Jordan, UT 84095, USA; okalloo@student.roseman.edu (O.R.K.); rlara1@student.roseman.edu (R.A.L.); mpavlova@roseman.edu (M.P.); 2 Department of Family Medicine and Public Health, Herbert Wertheim School of Public Health and Longevity Science, University of California San Diego, La Jolla, CA 92093, USA; dsilverstein@health.ucsd.edu; 3 Department of Family Medicine and Public Health, School of Medicine, University of California San Diego, La Jolla, CA 92093, USA

**Keywords:** COVID-19 pandemic, dental education, online learning, videoconferencing, alternative approaches to learning and teaching, comfort

## Abstract

**Background:** The COVID-19 pandemic caused many universities to expand their use of videoconferencing technology to continue academic coursework. This study examines dental students’ experience, comfort levels, and preferences with videoconferencing. **Methods:** Of 100 s-year US dental students enrolled in a local anesthesia course, 54 completed a survey following an online synchronous lecture given in August 2020. Survey questions asked about prior experience with videoconferencing, comfort levels with online and traditional classes, and reasons for not turning on their video (showing their face). **Results:** Overall, 48.2% had little or no experience with videoconferencing prior to March 2020. Students were more comfortable with in-classroom parameters (listening, asking questions, answering questions, and interacting in small groups (breakouts)) than with online synchronous learning, although differences were not significant (*p*’s > 0.10). Regression analyses showed there were significant positive associations between videoconferencing experience and comfort with both answering questions and interacting in breakouts (B = 0.55, *p* = 0.04 and B = 0.54, *p* = 0.03, respectively). Students reported being more comfortable during in-classroom breakouts than in breakouts using videoconferencing (*p* = 0.003). Main reasons for students not turning on their cameras were that they did not want to dress up (48.1%), other students were not using their video features (46.3%), and they felt they did not look good (35.5%). **Conclusions:** Dental students were somewhat more comfortable with traditional in-person vs. online classroom parameters. Prior experience with videoconferencing was associated with increased comfort with synchronous learning, suggesting that after the pandemic, it may be beneficial to structure dental school curricula as a hybrid learning experience with both in-person and online synchronous courses.

## 1. Introduction

The COVID-19 pandemic caused colleges and universities to move in an unprecedented way to online videoconferencing platforms such as Zoom, Microsoft Teams, and Google Meet to continue education. This remarkable technology gradually emerged in the years preceding the pandemic and, in 2020, exploded in growth [1]. For example, Zoom Video Communications, Inc. was developed in 2011 by Eric Yaun (San Jose, CA, USA) and launched in 2012 [2]. By May 2013, it had one million users; by September 2013, it had three million users; and by March 2020, Zoom’s daily meeting participants had grown to over two hundred million [3,4,5]. Zoom also topped the list of apps downloaded by iPhone users in 2020 [1,6,7].

This technology has the ability for one or more users to share their computer screen, send chat messages, organize small groups (breakouts), and record meetings. In short, it addresses the needs of students and teachers who are unable to meet in person [8]. As educational institutions embraced videoconferencing as an alternative to in-classroom education, it proved to be effective in certain regards. For instance, students generally felt that an advantage of videoconferencing was its flexibility and convenience [9,10].

However, several issues have arisen with the use of videoconferencing in dental education. A study of 1605 Turkish dental students reported that the majority (59.1%) felt that distance learning was not as effective as traditional face-to-face learning [11]. Likewise, a study of 31 US undergraduate students at a major university reported that while 22.6% enjoyed using Zoom, over twice as many students did not enjoy learning with this technology (48.4%) and 61.3% did not believe Zoom improved their learning [9].

Other negative effects of using videoconferencing in dental education have also been reported. For example, a study of US dental students and orthodontic residents at Roseman University reported that 43% found it often or always difficult to focus on schoolwork when courses were online [12]. Likewise, the majority of Turkish dental students surveyed reported that learning through Zoom had a negative effect on their ability to focus [11]. Furthermore, a study of 39 students at the Harvard School of Dental Medicine found a reported decrease in engagement and retention of material after switching from the traditional classroom to synchronous online learning due to COVID-19 [13].

This decreased engagement and focus may be associated with the fact that in some online environments, students can turn off their video feature and, in a sense, “be there, but not be there.” In other online environments, students are required to turn on their videos, which may be uncomfortable for some. In a study where students were asked about their comfort with using Zoom, the average response was neutral, indicating wide variation [9].

Comfort is defined as a state of psychological ease and experiencing no unpleasant feelings or thoughts due to nervousness or anxiety [14]. While others have examined students’ ability to focus as well as their engagement, retention, comfort, and perceived effectiveness of online learning, to our knowledge, no previous study has examined the factors that affect comfort levels of dental students using videoconferencing technology for dental education.

The purpose of this study is to (a) examine whether comfort levels of students using videoconferencing are associated with prior experience with videoconferencing; (b) compare students’ comfort level with videoconferencing to the traditional in-person classroom; and (c) provide insight into factors that prevent some students from using their video and engaging more fully in synchronous learning. 

## 2. Methods

### 2.1. Participants

This cross-sectional study was reviewed and approved by the Roseman University of Health Sciences Internal Review Board (IRB) and considered exempt (#1612317). Eligible participants were 100 s-year dental students enrolled in an online local anesthesia course at a US dental school located in the state of Utah, Roseman University College of Dental Medicine, from 15 July to 16 August 2020. Of 100 students eligible to participate, 54 responded to an electronic survey (response rate = 54%). This school began using online videoconferencing for all didactic courses on 16 March 2020.

### 2.2. Procedure

The didactic portion of the local anesthesia course consisted of both an asynchronous portion and a synchronous portion. Topics addressed during the synchronous part of the course included types of local anesthetic, needles, general principles of injections, supraperiosteal, middle superior alveolar, anterior superior alveolar, greater palatine, nasopalatine, and incisive and mental nerve blocks. The asynchronous portion consisted of fifteen brief videos and nine quizzes that students completed through Canvas, a learning management system, during a two-week period (15–30 July 2020).

The synchronous portion lasted approximately 3 h and 40 min, and was taught via Zoom on 3 August 2020. The professor let the students know several days beforehand that the lecture would have three breakout assignments, which were a required part of the course. He also let the students know that the lecture would be interactive and explained that students would be called on to answer questions. If the students did not feel comfortable being called on, they could email him beforehand. Only four students emailed requesting not to be called on during lecture. The synchronous lecture reviewed and expanded the concepts covered in the videos and quizzes.

A link to a survey created in Qualtrics was sent to students via email later in the same day as the synchronous lecture (3 August 2020), the day after lecture (4 August 2020), and three days after lecture (6 August 2020). The cover letter described the study and indicated that by completing the survey, students were giving their consent to participate. It was followed by twenty-two survey questions. Questions on the survey were designed to obtain information on demographic characteristics including age, gender (male/female/binary), and experience with videoconferencing prior to March 2020 (No experience/A little experienced/Somewhat experienced/Very experienced). A series of questions obtained information on student comfort levels in the traditional classroom, the virtual classroom (Zoom), and small breakout groups on a Likert scale from 1 to 7 with 1 = very uncomfortable and 7 = very comfortable. Students were also asked to rate their comfort level with the Zoom video feature (showing their faces) and to provide the reasons they did not show their faces. Students were asked to indicate the extent of their agreement that their comfort level increased as they gained more experience with the video feature using a 7-point Likert scale ranging from strongly disagree to strongly agree. Students were also asked to rate their engagement in the lecture when their video feature was on and whether their engagement increased when they were comfortable.

### 2.3. Statistical Analysis

Descriptive statistics including means as well as standard deviations for continuous variables and rates for categorical variables were calculated. Experience with videoconferencing prior to March 2020 was used as a continuous variable and as a categorical variable by dichotomizing it as little or no experience vs. somewhat or more experienced. Comparisons of those with little/none vs. some/very experienced on demographic characteristics were performed with independent *t*-tests for continuous outcome variables and with chi-square analysis for categorical variables. Comparisons of reasons for not using video, methods of communication during lecture and breakout sessions, and learning preferences by categorical experience were performed with chi-square analyses. Comparisons of agreement ratings with factors promoting engagement by categorical level of experience were performed with independent *t*-tests. 

Comparisons within students on mean comfort ratings with classroom parameters (listening, asking, and answering questions, and participating in breakout sessions) during online vs. traditional classrooms and when using the video feature (showing their face) were performed with paired *t*-tests. Linear regression analyses were used to examine the association of experience as a continuous variable with comfort ratings for in-person and online classroom activities, and for using the video feature. Sensitivity analyses were performed comparing comfort ratings with videoconferencing by gender and by age (categorized by median split) using independent *t*-tests. 

All statistics were performed with VassarStats [15], an online statistical computational program; *p* < 0.05 was considered statistically significant.

## 3. Results

The average age of participants was 27.7 years; 47.1% were females and 52.8% were males. Overall, 79.6% of students reported taking an online class before (see Table 1). Overall, 18.5% reported having no experience with videoconferencing prior to March 2020, 29.7% reported having a little experience, 33.3% reported being somewhat experienced, and 18.5% reported being very experienced. Comparisons by categorical experience showed that there were no significant differences between those with little/no experience vs. somewhat/very experienced with videoconferencing before March 2020 on age, gender, or history of having taken an online class (*p*’s > 0.05; see Table 1).

**Figure 1:**
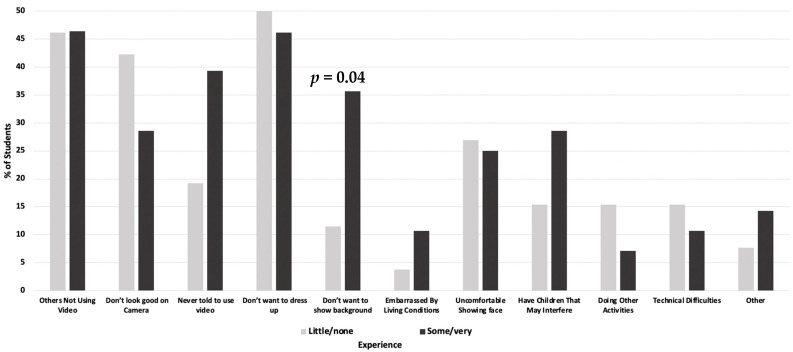
Comparisons of reasons for not using video by experience with video videoconferencing before March 2020 (N = 54). Comparisons performed with independent *t*-tests.

The most commonly reported reasons for not using the video feature in the online synchronous portion of the local anesthesia class were that students did not want to dress up (48.1%), other students were not using their video (46.3%), and they felt they did not look good (35.5%). An additional 22% did not use their video because they had children who they thought would interfere. Only 13.1% reported not using the video because of technical difficulties. There were 11% who reported not using the video because of doing other activities and 7.5% reported not using the video because they were embarrassed by their living conditions. As shown in Figure 1, compared to those less experienced with videoconferencing prior to March 2020, a significantly greater proportion of those who were more experienced reported not using the video feature because they did not want to show their background (means = 11.5 vs. 35.7, *p* = 0.04).

**Figure 2:**
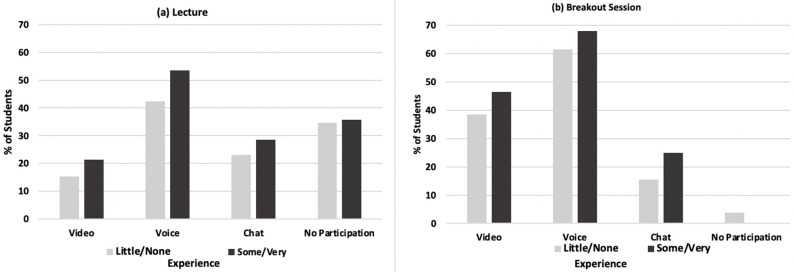
Comparisons of methods of communication used during (**a**) lecture and (**b**) breakout sessions by experience with videoconferencing before March 2020. Comparisons performed with independent *t*-tests.

Overall, the highest proportion of students reported using voice only to respond to questions during the synchronous anesthesia class (48.0%) and during the breakout sessions (64.7%). Over a third (35.2%) of all participants did not answer any questions during the synchronous lecture because they were not asked by the instructor. Comparisons of response methods used during the online synchronous lecture (Figure 2a) and during breakout sessions (Figure 2b) showed that there were higher rates of the use of video, voice, and chat for those with more experience, although differences were not significant (*p*’s > 0.10). Only 1.9% of all students did not participate in any breakout sessions and all were from those less experienced with videoconferencing prior to March 2020.

As shown in Table 2, compared to those less experienced, those more experienced with videoconferencing prior to March 2020 had significantly higher agreement with the statement that they were more engaged when they were comfortable with online learning (mean agreement ratings = 5.2 vs. 5.9, respectively, *p* = 0.03). Although those with more experience also had higher agreement with statements about being more engaged when their video was on or when knowing they might be asked a question, differences were not statistically significant (*p*’s > 0.10). There was also no significant difference in learning preferences by level of prior experience with videoconferencing (*p* > 0.10). 

Table 3 shows comparisons of mean comfort ratings with traditional in-person vs. online classroom parameters (listening, asking questions, answering questions, and interacting in breakout sessions). Students were significantly more comfortable interacting in breakout sessions during in-person than online classes (mean comfort ratings = 5.9 vs. 4.9, respectively, *p* = 0.003). For all other parameters (listening, asking questions, and answering questions), differences were non-significant (*p* > 0.10), although comfort ratings were always higher for in-person classes. Furthermore, although students gave higher comfort ratings for using the video feature during the anesthesia class than they did for March 2020, the difference was not significant (means = 4.7 vs. 4.1, respectively, *p* = 0.16).

Results of linear regression analyses examining the associations of experience with videoconferencing prior to March 2020 (as a continuous variable) and comfort levels for both in-person and online classroom activities, and with the video feature are shown in Table 4. There were significant positive associations between ratings of experience and comfort with both answering questions and interacting in breakouts (B = 0.55, *p* = 0.04 and B = 0.54. *p* = 0.03, respectively), indicating that ratings of comfort went up over half a point with each unit increase in the experience rating. Experience ratings also had significant positive associations with comfort with videoconferencing in March 2020 when students began using videoconferencing for online learning and when using videoconferencing for the online anesthesia class in August 2020. There were no significant associations of experience ratings with comfort listening to lectures or asking questions in the online classroom, or with comfort ratings for all in-person classroom activities (*p*’s > 0.05).

Sensitivity analyses showed that there were no significant differences by gender or by categorical age (younger vs. older) in mean comfort ratings for any of the in-person or online classroom activities (*p*’s > 0.10). There were also no significant differences by gender or by categorical age in mean comfort ratings using the video feature in March 2020 when using videoconferencing for the synchronous local anesthesia class or as they gained more experience (*p*’s > 0.10).

## 4. Discussion

The COVID-19 pandemic has altered education globally. It has forced universities, including professional schools, to move to online videoconferencing platforms to continue education. Although other researchers have reported that the majority of students are able to adapt to new technology [12], few previous studies have investigated students’ comfort levels during videoconferencing during online synchronous learning. This study showed that students were more comfortable with traditional in-person than online classroom parameters. It also showed that the amount of prior experience with videoconferencing was associated with increased comfort when asking or answering questions, as well as when interacting in breakout sessions during online classes. Additionally, students who reported more prior experience with videoconferencing were more comfortable using the video feature when they began using videoconferencing for courses in March 2020 and when using it for the online local anesthesia course in August 2020.

The results of this study are in accordance with previous studies [12,16,17] which found that students in professional schools were comfortable with using technology. For instance, a study of 1873 pharmacy students from 29 schools found that students were comfortable using technology, including videoconferencing, to communicate regarding coursework [17]. Adnan et al. [16] in a study of 126 undergraduate and postgraduate students taking an online course found that 61% reported feeling comfortable communicating through videoconferencing, although 67.5% reported feeling a difference between online learning and traditional in-classroom learning. Additionally, Hung et al. [12] found that among 145 dental students and orthodontic residents, 72% reported previously taking an online course and 87.6% reported high comfort adapting to technology in the online learning classroom. However, none of these studies examined the association of prior experience with comfort level and whether comfort level differed between online and traditional in-person courses.

In this study, students reported various reasons for not using the video feature during the synchronous portion of the online local anesthesia course. The most commonly reported reasons for not using the video feature were that they did not want to dress up, other students were not using their video, and they felt they did not look good. In this study, only 13.1% reported not using the video feature because of technical difficulties, a rate much lower than those reported in other studies. For instance, Candarli and Yuksel, [18] in a 2012 study of 36 higher education students, found that 42% reported not wanting to continue videoconferencing due to technical difficulties. Dost et al., [19] in a 2020 study of 2721 UK medical students, found that 21.5% reported that a poor internet connection was a barrier to online learning. Differences in the reliability of internet connections several years ago compared to now or between countries may account for these inconsistencies. Dost et al. [19] also reported that family distractions was described as a barrier to online learning by 26.8%, which is similar to the 22% rate reported in our study for the response choice of having children who might interfere with the online class. 

In this study, although not always statistically significant, students reported higher comfort ratings for the traditional in-person classroom than for the online classroom. This is in contrast to Dost et al. [19] who reported that medical students in the UK felt more comfortable with online learning, listing it as one of the advantages of online courses versus in-person courses. This difference may be due to variations in the medical vs. dental school curricula, as well as due to other educational differences between countries. In this study, comfort ratings for listening and for both asking and answering questions were relatively high, whether in-person or online. This is in contrast to Serhan [9] who reported that students’ average comfort rating with online learning was neutral. 

Earlier studies on gender and educational technology found significant gender differences with regard to access and performance outcomes that favored males and started as early as preschool [20,21]. Others reported that male secondary school students had more computer experience and showed more positive attitudes toward computers than female students [22]. However, recent studies report smaller differences between males’ and females’ adoption of technology or receptivity for distance learning [23]. Interestingly, in the present study, there were no significant differences between male and female students in their comfort level with using the technology or with having experience in videoconferencing prior to March 2020. Similarly, Ruthotto et al. [24] found a lack of significant gender differences in the participation in the virtual classroom among graduate computer science students. In contrast, others reported that gender and program level significantly affected the online learning experience for students. For instance, a study of online learning readiness in Malaysian university students [25] found that women were more ready than men to participate in online learning, were more satisfied, and had better learning experiences. Inconsistencies within the previous and current literature on gender differences in experience and comfort with educational technology may reflect generational and cultural differences rather than gender differences per se.

In this study, we also did not find any differences by age in comfort level with videoconferencing. This is in contrast to others who reported that younger students felt more comfortable using social media as a method to communicate about coursework than their older colleagues and/or fourth-year students [17]. Our results are also in contrast to those of a study of 1914 graduate students in computer science that reported that older students were more likely to participate in the online classroom than younger students and exhibited less “lurking” behavior, which is defined as the tendency to not participate and passively listen during a synchronous online course [25].

It is plausible that greater prior experience with videoconferencing is associated with higher levels of comfort when using this technology. According to the mere exposure effect [26], repeatedly exposing an individual to a stimulus enhances attitudes toward that stimulus. By increasing exposure, the stimulus becomes more familiar, increasingly predictable, and better liked [27]. Thus, increased prior exposures to videoconferencing may lead to more positive attitudes and comfort with it.

Benefits of incorporating a hybrid approach to teaching with both online synchronous and in-person classroom components include having students and faculty be better prepared to adapt to future emergency situations that may require synchronous online learning. It may also be a desirable teaching modality for circumstances requiring flexibility of both faculty and students, where travel to school for in-person classes is inconvenient or impractical [10]. Disadvantages, however, to offering both online synchronous and in-classroom components may include students relying on recordings, thus potentially decreasing attendance both in-person and online.

Solutions to the issues faced with videoconferencing in education may include providing continuous faculty development regarding methodologies and techniques to engage students when using online learning. Another solution may be for faculty to reduce the cognitive load in synchronous activities by increasing interactive activities as well as by providing quizzes and polls with videoconferencing [28]. Such initiatives may improve the engagement and comfort of both faculty and students using videoconferencing.

Several limitations and strengths of this study were considered. Data for this study came from students in a single course within a single US dental school. While this may limit generalizability, students in the sample may be comparable to other students pursing a doctoral degree in dentistry at other institutions. Furthermore, there was a wide age range (23 to 43 years) among the respondents and both genders participated in the survey. Additionally, the survey was conducted immediately after the online course had been completed, enabling respondents to better recall their comfort levels during the course. It would also be beneficial to examine the attitudes of students within several different courses to better understand student comfort in online synchronous learning versus the traditional classroom.

## 5. Conclusions

Results of this study suggest that after the pandemic, it may be beneficial to structure dental school curricula as a hybrid learning experience, with some traditional in-person courses and other courses or lectures offered through online synchronous videoconferencing. It may also be beneficial for faculty members to communicate expectations to students regarding videoconferencing protocols. We feel that these results would generalize to students at other dental schools and suggest less comfort with classes conducted via teleconferencing than in-person classes, even six months after the use of videoconferencing was mandated. Future studies should examine factors other than experience potentially contributing to student comfort such as engagement, motivation, and personal preference of the traditional classroom versus online learning.

## Figures and Tables

**Table 1:** Comparisons of demographic characteristics between those who were less vs. more experienced with videoconferencing before March 2020 (N = 54).

Characteristic	All	Experience		*t* or χ^2^	*p*
		Little/None (*n* = 26)	Some/Very (*n* = 28)		
**Age** (mean, sd)	27.7 (4.1)	28.6 (4.5)	26.9 (3.5)	1.53	0.13
Gender *					
Female (*n*, %)	25 (47.1)	11 (42.3)	14 (51.8)	0.18	0.67
Male (*n*, %)	28 (52.8)	15 (57.7)	13 (48.1)		
**Took online**					
**Class before**					
No (*n*, %)	11 (20.4)	6 (23.1)	5 (17.9)	0.02	0.89
Yes (*n*, %)	43 (79.6)	20 (76.9)	23 (82.1)		

* Note: One person in the somewhat/very experienced group described themselves as non-binary and was omitted from the gender analysis.

**Table 2:** Comparisons * of agreement ratings with factors promoting engagement and learning preferences between those who were less vs. more experienced with videoconferencing before March 2020 (N = 53).

Agreement Ratings—I Am More Engaged …	Experience		*t*	*p*
	Little/None (*n* = 25)	Some/Very (*n* = 28)		
	Mean	Mean		
When video is on (showing my face)	3.3	4.1	−1.62	0.11
Knowing I may be asked a question	4.8	5.3	−1.07	0.29
When I’m more comfortable with online learning	5.2	5.9	−2.23	0.03
	**%**	**%**	**χ^2^**	***p***
Learning preferences			3.56	0.31
Traditional in-person	32.0	28.6		
Online synchronous	24.0	17.9		
Both in-person and online synchronous	40.0	32.1		
Other	4.0	21.4		

* Comparisons performed with independent *t*-tests for agreement ratings and with chi-square analysis for learning preference. Response choices for agreement level were 1 = strongly disagree; 2 = disagree; 3 = slightly disagree; 4 = neutral; 5 = slightly agree; 6 = agree; and 7 = strongly agree.

**Table 3:** Comparisons * within students on mean comfort ratings with traditional in-person vs. online classroom parameters (N = 54).

Comfort with	In-Person	Online	*t*	*p*
Listening	5.9	5.8	0.37	0.71
Asking questions	5.1	4.8	1.10	0.28
Answering questions	4.8	4.6	0.47	0.64
Interacting in breakout sessions	5.9	4.9	3.08	0.003

* Comparisons performed with paired *t*-tests. Response choices for ratings of comfort level were 1 = very uncomfortable; 2 = uncomfortable; 3 = slightly uncomfortable; 4 = neutral; 5 = slightly comfortable; 6 = comfortable; and 7 = very comfortable.

**Table 4:** Associations * of experience with videoconferencing prior to March 2020 with comfort levels for in-person and online classroom activities, and with the video feature (N = 54).

Comfort Levels for…	Mean Comfort Rating	r	B	*p*
In-person classroom				
Listening to lectures	5.9	0.17	0.23	0.23
Asking questions	5.1	0.24	0.44	0.08
Answering questions	4.8	20	0.39	0.18
Interacting in breakouts	5.9	−0.02	−0.02	0.89
Online classroom				
Listening to lectures	5.8	0.18	0.30	0.21
Asking questions	4.8	0.25	0.48	0.07
Answering questions	4.6	0.28	0.55	0.04
Interacting in breakouts	4.9	0.30	0.54	0.03
**Comfort with video feature when…**				
Began using videoconferencing in March 2020	4.1	0.34	0.68	0.01
When using videoconferencing for online anesthesia class	4.7	0.26	0.52	0.05
As more experience was gained using videoconferencing	4.8	0.24	0.33	0.07

* Associations examined with Pearson correlations and linear regression analyses; B = slope or amount of change in comfort rating per unit increase in experience. Response choices for ratings of comfort level were 1 = very uncomfortable; 2 = uncomfortable; 3 = slightly uncomfortable; 4 = neutral; 5 = slightly comfortable; 6 = comfortable; and 7 = very comfortable.

## Data Availability

Data is available from the first author (D.G.M.).
